# High Expression of ITGA3 Promotes Proliferation and Cell Cycle Progression and Indicates Poor Prognosis in Intrahepatic Cholangiocarcinoma

**DOI:** 10.1155/2018/2352139

**Published:** 2018-02-04

**Authors:** Yu Huang, Yang Kong, Lufei Zhang, Tianyu He, Xiaohu Zhou, Yingcai Yan, Linshi Zhang, Dongkai Zhou, Sinan Lu, Jiarong Zhou, Lin Zhou, Haiyang Xie, Shusen Zheng, Weilin Wang

**Affiliations:** ^1^Key Laboratory of Precision Diagnosis and Treatment for Hepatobiliary and Pancreatic Tumor of Zhejiang Province, First Affiliated Hospital, School of Medicine, Zhejiang University, Hangzhou, China; ^2^Division of Hepatobiliary and Pancreatic Surgery, First Affiliated Hospital, School of Medicine, Zhejiang University, Hangzhou, China; ^3^Key Laboratory of Combined Multi-Organ Transplantation, Ministry of Public Health, First Affiliated Hospital, Zhejiang University School of Medicine, Hangzhou, China; ^4^State Key Laboratory & Collaborative Innovation Center for Diagnosis and Treatment of Infectious Diseases, First Affiliated Hospital, School of Medicine, Zhejiang University, Hangzhou, China

## Abstract

Integrin subunit alpha 3 (ITGA3) interacts with a beta 1 subunit to form a member of the integrin family. Integrins are heterodimeric integral membrane proteins that serve as cell surface adhesion proteins. In this research, we investigated the biological function of this protein in human intrahepatic cholangiocarcinoma (ICC) for the first time. Here, using Western blotting and immunohistochemistry assays, we discovered that ITGA3 was overexpressed in ICC cell lines and ICC patients. Moreover, we found ITGA3 expression correlated with several clinicopathological features, including tumor size, lymph node metastasis, and the TNM stage. Patients with high ITGA3 expression underwent a worse prognosis after complete resection compared with patients with low ITGA3 expression in terms of overall survival. Furthermore, we demonstrated that ITGA3 could significantly promote ICC cell proliferation and cell cycle progression* in vitro*. However, as a classical cell surface adhesion molecule, we found ITGA3 correlated negatively with the migration and invasion of ICC cell lines, which differs from other malignant tumors. Generally, these findings suggest that ITGA3 may play a role as a potential oncogene in ICC and suppression of ITGA3 expression may establish a novel target for guiding the therapy of ICC patients.

## 1. Introduction

Intrahepatic cholangiocarcinoma (ICC), deriving from the intrahepatic biliary tree, is the most common primary cancer of the liver ranking only second to hepatocellular carcinoma [1, 2]. As one of the most malignant human tumors, the surgical outcome of ICC is dismal [3, 4]. The median survival time for ICC patients who have lost the opportunity for an operation is usually less than six months, and the five-year survival rate for patients who undergo complete resection is only 20–40% [5–7]. Despite advances in surgical technology, little progress has been made in ICC therapy in recent decades [8, 9]. The awful prognosis of ICC patients is a result of the high possibility of recurrence and metastasis after complete surgical resection, in addition to resistance to systemic chemotherapy [10]. Thus, there is an urgent need to determine the molecular pathogenesis of ICC and to identify effective molecular targets to guide new treatments [11].

Integrins are heterodimeric integral membrane proteins that serve as cell surface adhesion proteins [12, 13]. ITGA3, a member of the integrin family, joins a beta 1 subunit to form an intact integrin and interacts with extracellular matrix proteins, including members of the laminin family. ITGA3 has been found to show aberrant expression in many malignant human tumors, including colorectal cancer, melanoma, and prostate cancer [14–18]. However, the function of ITGA3 in human ICC is still unknown. In this regard, we evaluated the clinical correlation and biological function of ITGA3 in human ICC for the first time.

## 2. Materials and Methods

### 2.1. Patients and Specimens

In total, 46 fresh ICC tumor samples and matched normal peritumor tissues were collected from patients who underwent resections of primary ICC in our department (Hepatobiliary and Pancreatic Surgery, The First Affiliated Hospital, College of Medicine, Zhejiang University, China) from 2013 to 2015. All the tissues were formalin-fixed and paraffin wax-embedded as soon as possible. No patient received preoperative therapy. The diagnosis of each specimen was checked by histopathology after the operation. Clinical data were collected and checked by two independent physicians, and the researchers were blinded to the clinical data. Overall survival was defined as the time from the date of the operation to death or recurrence. The study was approved by the Human Research Ethics Committee of The First Affiliated Hospital, College of Medicine, Zhejiang University. Written informed consent was obtained from all patients.

### 2.2. Cell Lines and Cell Culture

Hccc-9810 cells were purchased from the Chinese Academy of Sciences Shanghai Branch Cell Bank (Shanghai, China). The HuccT-1 cell line was maintained at our institution. The Hccc-9810 cell line was cultured in Roswell Park Memorial Institute (RPMI) 1640 medium (Gibco, Carlsbad, CA, USA) and the HuccT-1 cell line was cultured in Dulbecco's modified eagle medium (DMEM) (Gibco), supplemented with 10% fetal bovine serum (FBS; Gibco), penicillin (100 units/mL), and streptomycin (100 *μ*g/mL). All cells were maintained in a humidified atmosphere at 37°C containing 5% CO_2_ in an incubator and were passaged using standard cell culture techniques [19].

### 2.3. Transfection

HuccT-1 and Hccc-9810 were transfected with 50 nM siRNA (Life Technologies, Carlsbad, CA, USA) against ITGA3 using Lipofectamine 2000 (Invitrogen, Carlsbad, CA, USA) according to the manufacturer's protocol. The siRNA sequences were as follows: si1, 5′-GUGGGACUUAUCUGAGUAUTT-3′ (sense), 5′-AUACUCAGAUAAGUCCCACTT-3′ (antisense), si2, 5′-GGUGCCAUCUAUGUCUUCATT-3′ (sense), 5′-UGAAGACAUAGAUGGCACCTT-3′ (antisense), si3, 5′-GCACCUUCAUCGAGGAUUATT-3′ (sense), 5′-UAAUCCUCGAUGAAGGUGCTT-3′ (antisense), and siNC, 5′-UUCUCCGAACGUGUCACGUTT-3′ (sense), 5′-ACGUGACACGUUCGGAGAATT-3′ (antisense). Knockdown of ITGA3 was confirmed by Western blotting.

### 2.4. Immunohistochemistry

Sections of 3-*μ*m thickness were used for immunostaining. A rabbit polyclonal antibody against human ITGA3 (ab131055; Abcam, Cambridge, MA, USA) was used for immunohistochemistry (IHC) as described previously [19]. The IHC score was calculated by judging the percentage of positively stained cells. The scores ranged from 0 to 3 (0, 0%; 1, 5%; 2, 5–50%; and 3, >50% immunoreactive cells). Scores of 0 or 1 were considered to indicate “low” expression, while scores of 2 or 3 indicated “high” expression of ITGA3.

### 2.5. Cell Proliferation Assays

The effect of ITGA3 on cell viability was assessed by colorimetric immunoassay (Cell-Light EdU Apollo567 In Vitro Imaging Kit; Ribobio, Guangzhou, People's Republic of China) according to the manufacturer's instructions. Cells were cultured in a 20-mm confocal dish. The density of the cells is 1.5 × 10^5^ cells per dish for 24 h before transfection. The assay was performed 48 h after transfection.

For colony formation assays, cells transfected with siRNA were inoculated in 6-well plates at 1000 cells/well and maintained in a humidified atmosphere at 37°C containing 5% CO_2_, and the size of the colonies was observed after 2 weeks. Subsequently, the cells were fixed with methanol and stained with 1% crystal violet.

### 2.6. Transwell Assays

The migration and invasion assays were carried out in chambers of 8-*μ*m pore size (Corning, Corning, NY, USA) as described previously [20]. For migration assays, 4 × 10^4^ transfected cells in 200 *μ*L serum-free medium were added to the upper chamber, at the same time, 600 *μ*L DMEM with 10% FBS was placed in the lower chamber. The chambers were placed in 24-well plates and incubated at 37°C for 48 h. Subsequently, cells were stained with Diff-Quick stain (Polysciences, Warrington, PA, USA) according to the manufacturer's instructions. The cells were counted in ten fields using an inverted microscope (Leica, Malvern, PA, USA).

For invasion assays, 45 *μ*L of diluted Matrigel (1 : 8) was added to the upper chamber of the Transwell and after incubation at 37°C for 30 min, 1 × 10^4^ transfected cells in 200 *μ*L serum-free medium were added to the upper chamber. The remaining procedures of the invasion assay were the same as the migration assay.

### 2.7. Western Blot Analyses

Western blotting was performed as described previously [19]. Cells were washed by using cold PBS twice and then total proteins were extracted from the cells after incubating in a mixture of radioimmunoprecipitation assay (RIPA) lysis buffer (Cell Signaling Technology, Danvers, MA, USA) and complete protease inhibitor cocktail (Hoffman-La Roche Ltd., Basel, Switzerland) for 30 minutes on ice. After centrifugation (15,000 ×g, 4°C, 10 minutes), the supernatant was collected. A bicinchoninic acid protein assay kit (Thermo Fisher Scientific) was used to measure protein concentrations. For electrophoresis, equal amounts of denatured proteins were separated on 3%–8% NuPAGE Bis-Tris gels (Invitrogen, Carlsbad, CA, USA) at consistent 80 V for stacking gel about 1.5 h and 120 V for separating gel about 1 h. Then, the denatured proteins were transferred onto PVDF membranes at consistent 350 mA for 100 minutes. After blocking nonspecific binding for 1 h using 5% nonfat milk, the membranes were incubated overnight on ice with primary antibodies against GAPDH antibody (60004-1-Ig; Proteintech, NY, USA), ITGA3 (ab131055; Abcam, Cambridge, MA, USA), cyclin D1 (ab134175; Abcam), cyclin E1 (ab88259; Abcam), CDK2 (ab32146; Abcam), CDK4 (ab137675; Abcam), and CDK6 (ab124821; Abcam). Then the membranes were washed several times with TBST with vibration. Enhanced chemiluminescence detection was carried out after incubating with secondary horseradish peroxidase-labeled anti-mouse or anti-rabbit antibodies (1 : 2000) for 2 h at room temperature.

### 2.8. Cell Cycle Analysis

Cell cycle distribution was detected by flow cytometry. Before that, cells were fixed in 70% ethanol, stored at 4°C for more than 24 h, and stained with DNA PREP stain (Beckman Coulter, Brea, CA, USA).

### 2.9. Statistical Analysis

Data were analyzed using SPSS software (ver. 18; SPSS Inc., Chicago, IL, USA). All figures were performed by using GraphPad Software (GraphPad Prism® 6.0, La Jolla, CA, USA). The results are presented as mean ± SD. All experiments were performed in triplicate. *P* value < 0.05 was taken to indicate statistical significance.

## 3. Results

### 3.1. ITGA3 Is Overexpressed in ICC Tumor Specimens

**Figure 1:**
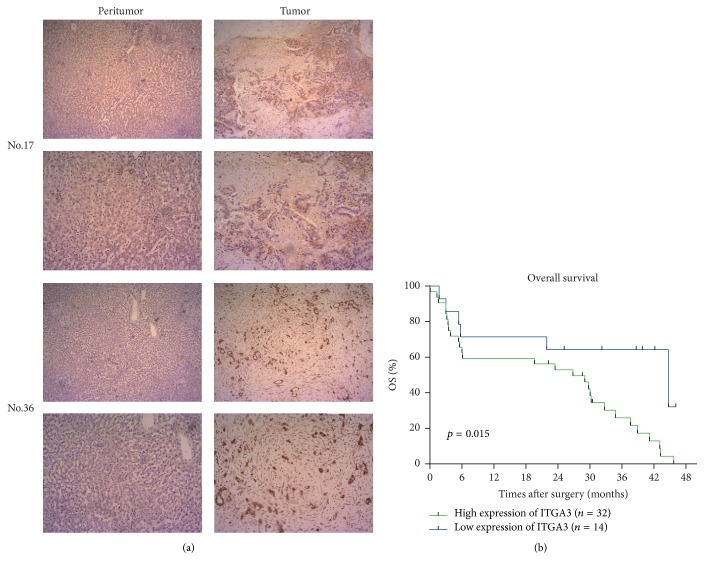
*Expression and clinical relationships of ITGA3 in ICC patients*. (a) Immunohistochemistry staining to detect ITGA3 expression in two representative paired human ICC specimens (tumor versus peritumor). Magnifications (×40 and ×100). (b) Survival analysis of ICC patients using Kaplan-Meier curves and log-rank tests. Patients were categorized by high (*n* = 32) and low (*n* = 14) expression of ITGA3, based on immunohistochemistry staining scores.* Abbreviations*. ICC: intrahepatic cholangiocarcinoma; ITGA3: integrin subunit alpha 3.

Immunohistochemistry assays were performed to analyze ITGA3 expression in paired tumor tissue slides and peritumor tissue slides obtained from 46 ICC patients from 2013 to 2015. We found that the expression of ITGA3 was higher in tumor tissues than in paired peritumor tissues of the 46-patient cohort. Two representative immunohistochemistry pictures of ICC patients are shown in Figure 1(a).

### 3.2. Association of ITGA3 Expression with Prognosis in ICC Patients

Survival analysis curves comparing the ICC patients with high and low ITGA3 expression in two patient cohorts showed that patients with relatively high ITGA3 expression (*n* = 32) had worse prognoses than those with lower ITGA3 expression (*n* = 14; *P* = 0.015; Figure 1(b)). Furthermore, a research of the relevance between the expression of ITGA3 and clinicopathological features in the 46-patient cohort showed that high expression of ITGA3 was significantly associated with large tumor size (*P* = 0.034), the presence of lymph node metastasis (*P* = 0.034), and advanced tumor stages (*P* = 0.027) (Table 1).

### 3.3. Expression of ITGA3 in ICC Cell Lines

**Figure 2:**
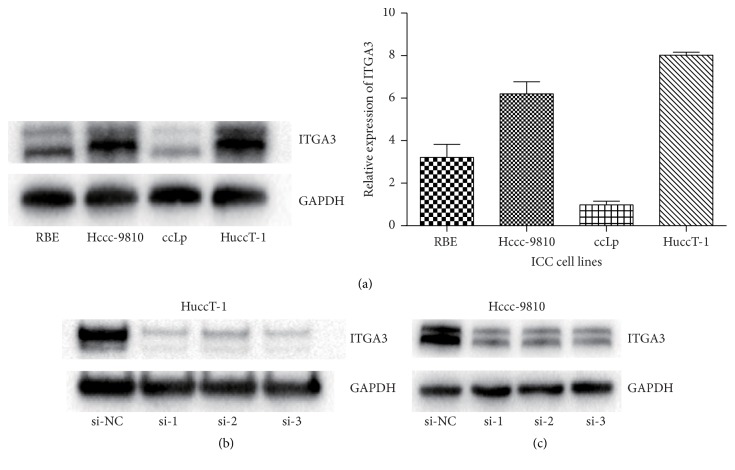
*Expression of ITGA3 in ICC cell lines*. (a) Western blot analysis and quantitative results for the relative expression of ITGA3 in ICC cell lines relative to GAPDH. (b) Identification of ITGA3 in HuccT-1 cells after transfection with siRNAs for ITGA3. (c) Identification of ITGA3 in Hccc-9810 cells after transfection with siRNAs for ITGA3. Si-1, Si-2, and Si-3 are three different siRNAs that affect different truncations.* Abbreviations*. ICC: intrahepatic cholangiocarcinoma; ITGA3: integrin subunit alpha 3; GAPDH: glyceraldehyde 3-phosphate dehydrogenase; NC: negative control; siRNA: short interfering RNA.

Recent studies have shown that high expression of ITGA3 correlated with poor prognosis in some tumors [16–18, 21]. Here, we also found that high expression of ITGA3 in ICC patients indicated a worse prognosis. To further examine the molecular pathogenesis of ITGA3 in ICC, we assessed ITGA3 expression in ICC cell lines with Western blotting. The results showed that the expression of ITGA3 was high in the ICC cell lines HuccT-1 and Hccc-9810 (Figure 2(a)). We also transfected HuccT-1 and Hccc-9810 cells with short interfering RNAs (siRNAs) for ITGA3. We constituted three different short interfering RNAs that influence different truncations of ITGA3 and named Si-1, Si-2, and Si-3. The results manifested that Si-3 was the most efficient and was used for the following experiments (Figures 2(b) and 2(c)).

### 3.4. ITGA3 Influences ICC Cell Proliferation

**Figure 3:**
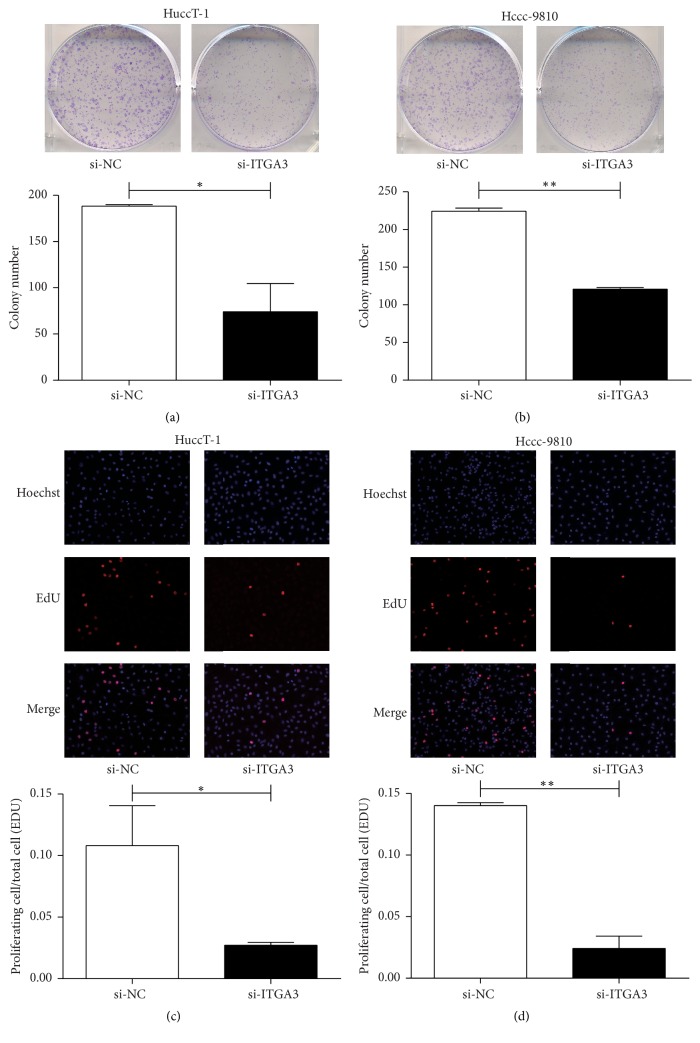
*Effect of ITGA3 on the proliferation of ICC cells*. (a) Colony formation assays and quantitative results in HuccT-1 cells after transfection with NC or ITGA3 siRNAs. (b) Colony formation assays and quantitative results in Hccc-9810 cells after transfection with NC or ITGA3 siRNAs. (c) EdU assays and quantitative results in HuccT-1 cells after transfection with NC or ITGA3 siRNAs. (d) EdU assays and quantitative results in Hccc-9810 cells after transfection with NC or ITGA3 siRNAs. Cells stained red indicate cells with high viability (^*∗*^*P* < 0.05, ^*∗∗*^*P* < 0.01; *n* = 3). Magnification ×200. *P* values were determined with paired *t*-test.* Abbreviations*. ICC: intrahepatic cholangiocarcinoma; ITGA3: integrin subunit alpha 3; NC: negative control; siRNA: short interfering RNA; EdU: 5-ethynyl-2′-deoxyuridine.

A colony formation assay showed that knockdown of ITGA3 restricted colony formation in HuccT-1 and Hccc-9810 cells, respectively (Figures 3(a) and 3(b)). The EdU assay results showed that silencing ITGA3 suppressed the proliferation of HuccT-1 and Hccc-9810 cells, consistently with the colony formation assay (Figures 3(c) and 3(d)).

### 3.5. ITGA3 Promotes ICC Cell Cycle Progression

**Figure 4:**
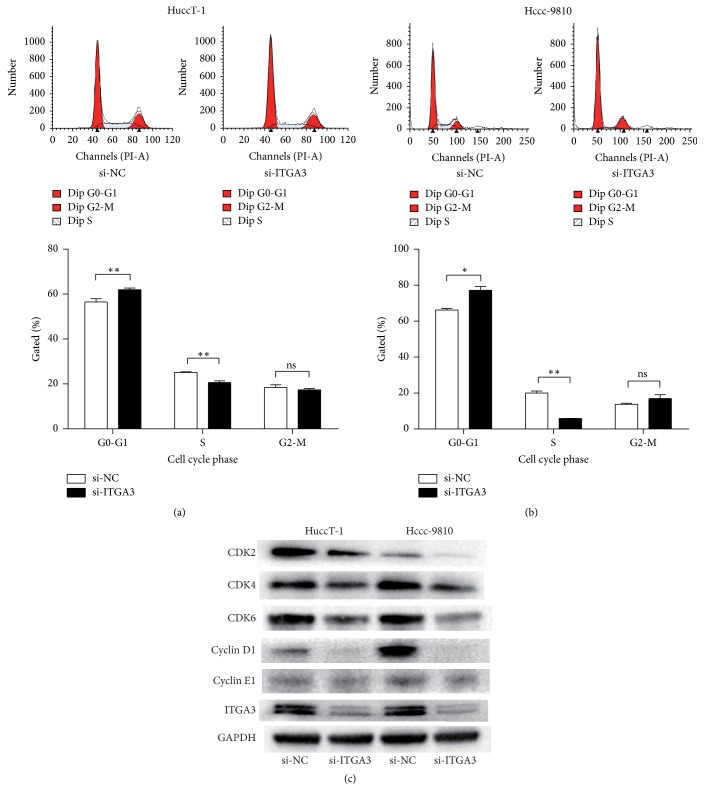
*ITGA3 regulates cell cycle progression in ICC cell lines*. (a) Representative fluorescence-activated cell sorting (FACS) images and quantitative results for HuccT-1 cells treated with NC or ITGA3 siRNAs. (b) Representative FACS images and quantitative results for Hccc-9810 cells treated with NC or ITGA3 siRNAs. ^*∗*^*P* < 0.05, ^*∗∗*^*P* < 0.01, ns: not significant; *n* = 3. (c) Effect of ITGA3 on the expression of cyclin and cyclin-dependent kinase (CDK) proteins by Western blot assay. *P* values were determined with paired *t*-test.* Abbreviations*. FACS: fluorescence-activated cell sorting; NC: negative control; ICC: intrahepatic cholangiocarcinoma; ITGA3: integrin subunit alpha 3; siRNA: short interfering RNA.

Considering the results above, that ITGA3 affected the ability of ICC cells to proliferate significantly, we next analyzed changes in the ICC cell cycle induced by silencing ITGA3 expression. Knockdown of ITGA3 influenced the cell cycle in HuccT-1 and Hccc-9810 cells significantly. Cells were arrested in G1 phase and fewer entered into S phase, compared with cells transfected with the negative control (NC). Cells in G2 phase showed no significant difference (Figures 4(a) and 4(b)).

We then considered whether the proteins regulating the cell cycle were altered. SiRNA knockdown of ITGA3 downregulated the levels of cyclin-dependent kinase (CDK) 2, CDK4, and CDK6 significantly, as well as cyclin D1 and E1 in HuccT-1 and Hccc-9810 cells (Figure 4(c)).

### 3.6. ITGA3 Expression Negatively Correlates with the Migration of ICC Cells

**Figure 5:**
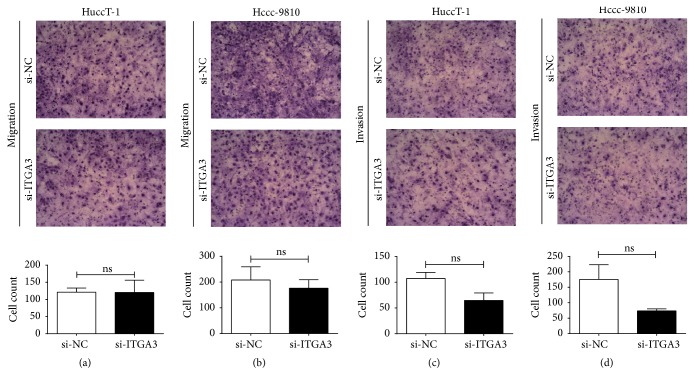
*ITGA3 expression negatively correlated with migration and invasion in ICC cells*. (a) Cell migration assays and quantitative results for HuccT-1 cells transfected with NC or ITGA3 siRNAs. (b) Cell migration assays and quantitative results for Hccc-9810 cells transfected with NC or ITGA3 siRNAs. (c) Cell invasion assays and quantitative results for HuccT-1 cells transfected with NC or ITGA3 siRNAs. (d) Cell invasion assays and quantitative results for Hccc-9810 cells transfected with NC or ITGA3 siRNAs. ns: not significant; *n* = 3. Magnification ×200. *P* values were determined with paired *t*-test.* Abbreviations*. ICC: intrahepatic cholangiocarcinoma; ITGA3: integrin subunit alpha 3; NC: negative control; siRNA: short interfering RNA.

Previous studies have reported that the epithelial-mesenchymal transition is a major process that provides cancer cells with increased abilities of migration and invasion, which causes the metastasis of malignant tumors [22–24]. As a classic cell surface adhesion molecule, ITGA3 expression has been reported to correlate positively with the metastasis of many human tumors [14–17]. Considering the relevance between the expression of ITGA3 and clinicopathological features in ICC patients, the ICC cell lines HuccT-1 and Hccc-9810 were transfected with siRNA* in vitro* and used for Transwell experiments to further explore the relevance between ITGA3 expression and the metastatic ability of ICC cells. However, there was no significant difference between the siRNA and the NC group, indicating that the overexpression of ITGA3 was not involved in the invasion or migration of ICC cells* in vitro* (Figures 5(a)–5(d)), which was not consistent with other malignant tumors [14–17]. Klinowska et al. reported that mammary gland epithelial development and differentiation were not dependent on alpha 3 integrin subunit [25]. At this point, we suggest that ITGA3 is involved in different oncogenic pathways because of different tumors' different locations, compositions, and microenvironments. Furthermore, due to the different environment of ICC cells* in vivo* and* in vitro*, some biological characteristics might change. In summary, other molecular mechanisms where ITGA3 participates in the metastasis of ICC are still valuable to explore.

## 4. Discussion

Increased data have revealed that ITGA3 expression levels positively correlate with the prognosis of many malignant human tumors, including colorectal and prostate cancers [14, 15]. Although it has been investigated in different kinds of human tumors, our research has demonstrated an underlying oncogenic role of ITGA3 in ICC for the first time. In this study, we determined that ITGA3 was overexpressed at the protein level in ICC cell lines and patients. Furthermore, we investigated the relations between ITGA3 expression and some clinicopathological features of ICC patients. We discovered that aberrant overexpression of ITGA3 in ICC patients is related to some malignant clinicopathological characteristics, such as gross tumor size, lymph node metastasis, and TNM stage. Moreover, survival curve analyses indicated that ICC patients with higher ITGA3 expression underwent worse outcomes. In conclusion, our findings suggest that ITGA3 expression may be used as a promising predicted molecular marker for disease outcome of ICC patients.

*In vitro*, ITGA3 was overexpressed at the protein level in ICC cell lines HuccT-1 and Hccc-9810. The overexpression of ITGA3 accelerated cell proliferation and cell cycle progression in ICC cells compared with the controls. Thus, ITGA3 overexpression becomes a prediction of a more malignant grade of ICC. Nevertheless, as a cell surface adhesion molecule, ITGA3 correlated negatively with the migration and invasion of ICC cells* in vitro* in our study, which seemed paradoxical. We consider that ITGA3 may play diverse roles through different oncogenic pathways in various malignant human tumors because of the tumors' derivation, location, and surrounding microenvironment. Moreover, due to the differing environments of ICC cells* in vivo* and* in vitro*, some cell biological characteristics may change. In this regard, further exploration will be valuable to identify how ITGA3 functions to promote the metastasis of ICC to guide treatment.

Due to the lack of specific effective neoadjuvant therapies for ICC, conventional radical surgery remains the only way to “cure” ICC patients [26, 27]. However, despite advances in surgical technology and emerging adjuvant treatments in recent decades, the prognosis for ICC patients after radical resection remains dismal, due to the high recurrence rate [8, 9]. In our study, we discovered that aberrant high expression of ITGA3 correlated with proliferation and poor prognosis in ICC patients, indicating that ITGA3 overexpression accelerates tumor progression in ICC. We assessed whether ITGA3 expression may be used to provide a classification of ICC patients. Additionally, suppression of ITGA3 may supply a promising new guideline for the treatment of ICC patients.

## 5. Conclusion

This research is the first reported demonstration that ITGA3 is overexpressed and functions as an oncogene in the proliferation and cell cycle of ICC cell lines, which correlated with tumor progression in ICC patients. The clinical correlation and function of ITGA3 in ICC make it a promising diagnostic and therapeutic target for drug research and development in the future.

## Figures and Tables

**Table 1:** Correlation between ITGA3 expression and clinicopathological features of ICC patients.

Clinical parameter	ITGA3 expression		*P* value
	High	Low	
Age (years)			
≥60	19	9	0.754
<60	13	5	
Gender			
Male	17	7	0.845
Female	15	7	
HBV			
Yes	7	3	1.000
No	25	11	
AFP			
≥20 *µ*g/L	5	0	0.303
<20 *µ*g/L	27	14	
CA19-9			
≥37 U/ml	26	11	1.000
<37 U/ml	6	3	
Tumor size			
≥6.5 cm	20	4	**0.034**
<6.5 cm	12	10	
Tumor number			
Single	17	10	0.246
Multiple	15	4	
Lymph node metastasis			
Yes	20	4	**0.034**
No	12	10	
Liver cirrhosis			
Yes	11	5	1.000
No	21	9	
TNM stage			
I-II	5	7	**0.027**
III-IV	27	5	

Statistical analyses by Pearson's *χ*^2^ test or Fisher's exact test. *Abbreviations*. ICC: intrahepatic cholangiocarcinoma; ITGA3: integrin subunit alpha 3. Statistically significant values are boldfaced.
